# Rapid Review of SARS-CoV-1 and SARS-CoV-2 Viability, Susceptibility to Treatment, and the Disinfection and Reuse of PPE, Particularly Filtering Facepiece Respirators

**DOI:** 10.3390/ijerph17176117

**Published:** 2020-08-22

**Authors:** José G. B. Derraik, William A. Anderson, Elizabeth A. Connelly, Yvonne C. Anderson

**Affiliations:** 1 Liggins Institute, University of Auckland, Auckland 1023, New Zealand; 2 Department of Paediatrics, Child and Youth Health, University of Auckland, Auckland 1023, New Zealand; y.anderson@auckland.ac.nz; 3 Tamariki Pakari Child Health and Wellbeing Trust, New Plymouth, Taranaki 4310, New Zealand; 4 Department of Women’s and Children’s Health, Uppsala University, 751 85 Uppsala, Sweden; 5 Department of Chemical Engineering, University of Waterloo, Waterloo, ON N2L 3G1, Canada; wanderson@uwaterloo.ca; 6 Dermatology, Department of Medicine, Taranaki District Health Board, New Plymouth 4310, New Zealand; lisa.connelly@tdhb.org.nz; 7 Department of Paediatrics, Taranaki District Health Board, New Plymouth 4310, New Zealand

**Keywords:** coronavirus, COVID-19, decontamination, disinfection, filtering facepiece respirators, heat, N95, personal protective equipment, reuse, SARS-CoV-1, SARS-CoV-2, viability, temperature, ultraviolet light, UVC

## Abstract

In the COVID-19 pandemic caused by SARS-CoV-2, hospitals are often stretched beyond capacity. There are widespread reports of dwindling supplies of personal protective equipment (PPE), particularly N95-type filtering facepiece respirators (FFRs), which are paramount to protect frontline medical/nursing staff, and to minimize further spread of the virus. We carried out a rapid review to summarize the existing literature on the viability of SARS-CoV-2, the efficacy of key potential disinfection procedures against the virus (specifically ultraviolet light and heat), and the impact of these procedures on FFR performance, material integrity, and/or fit. In light of the recent discovery of SARS-CoV-2 and limited associated research, our review also focused on the closely related SARS-CoV-1. We propose a possible whole-of-PPE disinfection solution for potential reuse that could be rapidly instituted in many health care settings, without significant investments in equipment.

## 1. Introduction

In pandemic situations, such as the ongoing COVID-19 pandemic, hospital resources are frequently stretched beyond capacity, as has already occurred in many countries across the globe [1]. Preventing the spread of COVID-19 to and from health care workers and patients relies on the availability and effective use of personal protective equipment (PPE) [1]. PPE includes masks, eye protection, gloves, gowns, and, for aerosol-generating procedures in particular, N95 filtering facepiece respirators (FFRs) or equivalent [2].

There has been a global shortage of PPE during the current pandemic [3], and the World Health Organization acknowledges the current global stockpile has been insufficient, especially for surgical masks and FFRs [2]. The supply of gowns and eye protection is also expected to be insufficient. Coordinating the supply chain of PPE in the midst of a pandemic with many closed borders and reduced freight is challenging. Individual behavior becomes a factor when people are scared or ill-informed [4]. Ideally, people need to have trust in the systems set up to support them in the workplace [5]. When this trust is compromised and local supply chains are affected, inappropriate use of PPE can occur, sometimes with theft of PPE further affecting supply, despite best-practice guidance on its use [2]. This shortage of PPE in the face of an exponential increase in demand also seems to have encouraged the production of counterfeit FFRs, potentially creating additional risks for health care workers [6]. Unsurprisingly, a call for ideas on conserving PPE was made through the Journal of the American Medical Association (JAMA) in March 2020 [7].

One key recommendation to deal with the unprecedented shortage of PPE has been disinfecting and reusing PPE, particularly FFRs [8]. It is acknowledged that PPE items are designed for single use. However, the reality during the course of the pandemic is that reuse has been undertaken by many health care workers across the world out of necessity [9]. Therefore, understanding how to effectively disinfect PPE items for potential reuse was the focus of this study. Given the ability of PPE decontamination and reuse to rapidly address supply issues close to the “frontline” (thus avoiding many of the upstream disruptions to the supply chain), we carried out a rapid review to summarize the relevant literature with three specific aims—first, to examine the current knowledge about the viability of SARS-CoV-2 on a variety of surfaces; second, to determine the efficacy of key disinfection procedures against SARS-CoV-2, specifically ultraviolet light and heat; and third, to determine the impact of these procedures on FFR performance. In light of the very recent discovery of SARS-CoV-2, our review also focused on SARS-CoV-1, a closely related sister clade virus from the same species [10]. Further, based on current knowledge, a possible whole-of-PPE solution for potential reuse is suggested that could be rapidly instituted in many health care settings without significant investments in equipment.

## 2. Methods

We carried out a rapid review, as it can provide valuable information for decision-making in a timely manner, particularly important in a pandemic scenario. There is no consensus in the literature for either the definition of a rapid review or the most appropriate methodology [11]. Nonetheless, our search strategy involved PubMed, Web of Science, and Google Scholar (in this order).

Searches were restricted to publications from 1 January 2003 (as the first recorded human infection of SARS-CoV-1 occurred in November 2002 [12]) and 18 July 2020. A number of keywords were used alongside the term “SARS” in combination with Boolean operators (Table 1).

The results from the literature search (Table 1) had their title and/or abstract screened by the first author, and those deemed to be of relevance were immediately exported to a bibliographical software, and the respective full text subsequently obtained. Where appropriate, other articles were included, if discovered while examining the full text of individual studies. Any original study reporting quantitative data addressing any one of the three aims of this rapid review was included for data extraction. However, in the context of this rapid review, we acknowledge as a limitation the lack of a formal assessment of the evidence quality of the included studies.

### Filtering Facepiece Respirators

This rapid review has focused in particular on FFRs, which remain at the center of the PPE shortage worldwide. There is conflicting evidence on the superiority of FFRs over standard surgical masks to protect frontline staff against viral respiratory infections during standard care [13,14,15,16,17]. However, approximately 3.2% of patients with SARS-CoV-2 in China required intubation during the first wave [18], and evidence from the SARS epidemic showed that doctors and nurses involved in the early critical care period and endotracheal intubation of patients were over 13 times more likely to acquire SARS-CoV-1 infection themselves [19]. Thus, FFRs are particularly important for health care workers during aerosol-generating procedures in patients with SARS-CoV-2. Lack of adequate protection could result in a significant loss of highly specialized health care workers in an already strained workforce, exacerbating community transmission. Therefore, avoidance of cross contamination is critical in all health care settings, and FFRs play a key role.

We note that while the term “N95” has achieved global reach, it actually refers to the US National Institute for Occupational Safety and Health (NOISH) certification [20,21]. N95 FFRs are defined as respirators not resistant to oils, but with a particle filtration efficiency ≥95% when challenged with sodium chloride particles of a median diameter of 0.075 μm at a flow rate of 85 L/min [20,22]. The equivalent Conformité Européen (CE) certifications are FFP2 and FFP3 respirators, which have minimum required particle filtration efficiencies of 94% and 99%, respectively [21]. Thus, we have referred to FFRs instead of N95 throughout this manuscript, whenever referring to this generic group of respirators.

## 3. Virus Viability

During the SARS epidemic, SARS-CoV-1 was recovered from a variety of inanimate objects and surfaces, e.g., buttons of drinking water fountains, chairs, bookshelves, tables, and edges of a bed [23]. Two small studies from the same group in Singapore showed no SARS-CoV-2 contamination on PPE after some contact with infected patients [24,25], although none of those patients required ventilation support or aerosol-generating procedures. Nonetheless, SARS-CoV-1 has been recovered from door handles in a patient’s room [26] and SARS-CoV-2 from uncovered shoes [24]. Such observations led the author of the former study to speculate that virus contamination of these surfaces may have led to infection among health care workers without documented contact with known hospitalized SARS patients [23]. As a result, it is important to understand the viability of SARS-CoV-2 on a variety of surfaces, particularly due to its relevance for PPE and health care/frontline worker protection.

### 3.1. SARS-CoV-1

The available evidence on the viability of SARS-CoV-1 has been summarized from nine studies in Table 2. Materials tested included cardboard [27], wood [28,29], plastic [27,28,29,30,31,32], fabric [28,29,30], paper [28,29,30], glass [28,29,33,34], and metal [27,28,29] (Table 2). Virus viability on the range of materials tested varied markedly, even within type (e.g., stainless steel vs. copper [27]). For example, Li et al. [29] reported a ~4.0 log_10_ reduction in cloth material after 6 h but it took 48 h to achieve a ~2.0 log_10_ reduction on wood (Table 2). One study looking specifically at PPE demonstrated viability of 2 days on a disposable polypropylene gown (6.0 log_10_ reduction) and 24 h on a cotton gown (6.0 log reduction) [30] (Table 2).

Of note, SARS-CoV-1 remained infectious at room temperature for as long as 9–10 days on plastic petri dishes [31] and on respiratory specimens [30], and 21 days on plastic well plates [32], with limited loss of infectivity after 2 weeks at 4 °C shown by at least two other studies [33,35] (Table 2). However, Pagat et al. [34] showed a 2.5 log_10_ TCID_50_/mL viral titre reduction in the first day when the inoculum passed from liquid to dried form, but afterwards it took as many as 42 days for complete inactivation of SARS-CoV-1 (~6.2 log_10_ reduction) at room temperature on a glass surface (Table 2).

It is important to highlight the effect of the virus load and inoculum size on SARS-CoV-1 inactivation, as reported by Lai et al. 2005 [30]. While inoculation of a cotton gown with 4 log_10_ TCID_50_/mL led to inactivation in 5 min, inactivation took 24 h with 6 log_10_ TCID_50_/mL.

### 3.2. SARS-CoV-2

Six peer-reviewed studies and one non-peer-reviewed study have examined the viability of SARS-CoV-2 (Table 3). Virus viability was assessed on a number of different materials, including glass [33,36,37], metal [27,36,37,38,39,40], plastic [27,37,38,40], paper [37], cardboard [27], rubber [38,40], wood, and cloth [37] (Table 3). As observed for SARS-CoV-1, there was marked variation in the viability of SARS-CoV-2 across different types of materials (Table 3). For example, Chin et al. [37] reported a ~3.5 log_10_ reduction in SARS-CoV-2 virus titre after 3 h on tissue paper, but it took 4 days to achieve a ~3.8 log_10_ reduction on glass (Table 3).

In addition, two studies [33,37] showed that lower temperatures are more favorable for SARS-CoV-2 viability, with little virus titre decay shown after 2 weeks at 4 °C (Table 3). However, even at room temperature (20–25 °C), the two studies showed that it can take 14 days to achieve a 4.5–5.0 log_10_ reduction of SARS-CoV-2 in applied virus droplets (Table 3).

Importantly, some studies have determined the viability of SARS-CoV-2 directly on PPE or PPE-derived materials, including FFRs [37,38,39,40] (Table 3). Fischer et al. [39] reported a ~4.0 log_10_ reduction of SARS-CoV-2 on N95 FFR disks after 24 h but, according to Chin et al. [37], it took 7 days to achieve ~4.8 log_10_ and ~3.0 log_10_ reductions in SARS-CoV-2 infectivity on the inner and outer layers of surgical masks, respectively (Table 3). Notably, while yet to be peer reviewed, the study by Kasloff et al. [40] assessed SARS-CoV-2 viability using a soil load of mucin, bovine serum albumin, and tryptone as the inoculum, said to represent typical infectious body fluids of infected patients. That study showed that to achieve a 5.0 log_10_ reduction in SARS-CoV-2 at room temperature (~20 °C), it took as long as 14 days on nitrile rubber gloves and 21 days on plastic face shields, N100 respirators, and polyethylene coveralls, with some residual infectivity still remaining on N95 respirators after 3 weeks [40] (Table 3).

## 4. Disinfection

A wide variety of potential disinfection methods for PPE have been examined and reported in the literature. These can be characterized as: (1) energetic methods (e.g., ultraviolet, dry and moist heat, and microwave generated steam), or (2) chemical methods (e.g., alcohol, ethylene oxide, bleach, and vaporized hydrogen peroxide). Some of these rapidly and markedly affect N95 particle filtration performance (alcohol [22,41,42,43,44]), while others require chemical supplies and/or specialized facilities (e.g., ethylene oxide and vaporized hydrogen peroxide), or are not readily scalable to large numbers of PPE (e.g., microwave generated steam). Therefore, the focus of this review is on methods that may be easy to implement rapidly at large scale.

### 4.1. Ultraviolet Germicidal Irradiation (UVGI)

Across the ultraviolet (UV) light spectrum that is invisible to the human eye, for the purposes of this review, there are three classifications according to wavelength: UVA (320–400 nm), UVB (280–320 nm), and UVC (200–280 nm) [45]. UVC light has much stronger germicidal properties than both UVA and UVB [46,47]. UVC is strongly absorbed by RNA and DNA bases, leading to molecular structural damage via a photodimerization process; this results in virus inactivation, as the virus is no longer able to replicate [47,48]. Thus, for PPE decontamination, the focus is on UVC rather than UVA or UVB. There have been seven studies assessing the efficacy of UVGI against SARS-CoV-1 and six against SARS-CoV-2, although one of the latter was reported in such a way that was not deemed to be of relevance (Table 4).

For SARS-CoV-1, the applied dose of ultraviolet light C (UVC) used varied markedly from 300 to 14,500 mJ/cm^2^, with rather mixed outcomes (Table 4). At the lower end of the spectrum, a ~6 log_10_ reduction in virus titre was achieved in culture medium with 300 mJ/cm^2^ [28] (Table 4). Conversely, in a high-protein solution, an applied dose of 14,500 mJ/cm^2^ did not completely inactivate SARS-CoV-1 [45] (Table 4), most likely due to competitive absorption of UV photons by the protein medium. Of note, a number of these studies were performed on culture media or under conditions that would not reflect the micro-environments likely to be found in FFRs or other PPE destined for disinfection and reuse in a real-world setting. As a result, some of these findings need to be interpreted in the appropriate context and be seen as guidance. Nonetheless, Heimbuch and Harnish (2019) showed complete inactivation (≥4.0 log_10_ reduction) of SARS-CoV-1 from FFR coupons in the presence of artificial saliva (mucin) and artificial skin oil (sebum) with an applied UVC dose of 1000 mJ/cm^2^ [51].

There were few available UVGI studies on SARS-CoV-2 (Table 4). Heilingloh et al. [54] inactivated SARS-CoV-2 in a liquid medium (>6.7 log_10_ reduction) with an applied UVC dose of 1048 mJ/cm^2^ (Table 4). Fischer et al. [39], Smith et al. [42], and the unpublished Ozog et al. [56] studied inactivation on N95 respirators, and UVGI treatment was not entirely efficacious against SARS-CoV-2. In Ozog et al., not all samples tested achieved a ≥3.8 log_10_ reduction with UVC at 1500 mJ/cm^2^, with treatment failure common on FFR straps [56] (Table 4). In Fischer et al. [39], while a ≥4 log_10_ reduction was achieved against SARS-CoV-2 on stainless steel with an applied UVC dose of 330 mJ/cm^2^, on N95 samples 1980 mJ/cm^2^ did not lead to a reduction in virus titre greater than approximately 3 log_10_ (estimated from their figure) (Table 4).

While there is no doubt that UVC is effective against both SARS-CoV-1 and SARS-CoV-2, efficacy of the applied dose (a function of irradiance and time) appears to be highly dependent on many factors, such as virus titre, inoculum size, the virus medium, and both shape, contours and type of material [30,47,51,58], likely explaining the highly inconsistent findings in the published literature. However, based on the available evidence, it seems that the effect of relative humidity on UVGI efficacy can be considered negligible [59].

Importantly, the applied dose is not necessarily the same as the actual dose the target virus receives at any specific point. While the applied dose is easy to measure experimentally using a radiometer, the received dose at each microscopic location is not. If there are shadowing or absorption effects from the surrounding medium, structures, or surface irregularities, the actual dose reaching the virus will be lower [60,61]. In addition, the penetration of UV across the multiple layers of an N95 FFR may vary from one model and manufacturer to another [62]. There is some limited evidence that the majority (approximately 90%) of captured aerosols occurs on the outer filter layer on an N95 FFR [63], but good disinfection efficacy is still desirable across all the layers.

### 4.2. Heat Treatment

Heat treatment is one of the most common methods utilized for disinfection, including for virus deactivation. Heat induces structural changes in virus proteins, disrupting the specific structures necessary to recognize and bind to host cells [64]. The challenge for heat treatment is to eliminate the virus without damaging PPE, in particular FFRs. Eight studies were found to have examined the efficacy of heat treatment against SARS-CoV-1 and eight against SARS-CoV-2 (Table 5). 

It should be noted that it is not easy to extrapolate the results from most heat treatment studies reported here. They were often performed with the virus exposed while in solution, which is mechanistically different from surface contamination, as one would most likely encounter on PPE that is not heavily soiled, particularly FFRs.

Environments with lower temperatures seem to be more favorable for virus viability and increased transmission rates [32,71,72], which also applies to SARS-CoV-1 and SARS-CoV-2 (Table 2). While the efficacy of heat treatment appears to be affected by relative humidity [32], this relationship for both viruses of interest here is unclear, as almost all experimental studies failed to report on this parameter (Table 5). However, the association between temperature and relative humidity was not monotonic for other coronaviruses, with virus survival lowest at moderate relative humidity (50%) [72].

Heat treatment at 56 °C reduced SARS-CoV-1 virus titre below levels of detection after 20 min (~4.2 log_10_ reduction) [45], 30 min (~8 log_10_ [35] and ≥5.0 log_10_ [31]), 60 min (≥6.4 log_10_) [50], and 90 min (≥4.0 log_10_ [28] and ~5.0 log_10_ [46]), noting that Rabenau et al. showed that a protein medium adversely affected the efficacy of heat treatment at this temperature against the virus [31] (Table 5). At 60 °C, SARS-CoV-1 was inactivated after 30 min (~3.0 to 5.5 log_10_ reduction depending on plasma product [65], ≥5.0 log_10_ [31], and ≥3.5 log_10_ [45]). Not surprisingly, faster viral inactivation was achieved at higher temperatures, with, for example, reductions of ≥4.0 log_10_ [46] and ~4.2 log_10_ [45] achieved after 5 and 10 min at 65 °C, respectively (Table 5).

For SARS-CoV-2, after 30 min at 56 °C, the magnitude of the virus titre reduction was 3.0 log_10_ [33], ≥4.6 log_10_ [37], ≥5.0 log_10_ [66], ≥5.9 log_10_ [67], 5.0–6.0 log_10_ [69], and 7.0 log_10_ [70]. At 60 °C, there was a 7.0 log_10_ reduction after 15 min [70], and a 5.0–6.0 log_10_ reduction (complete inactivation) in clinical samples after 60 min [69] (Table 5). According to an unpublished study [67], at 65 °C, SARS-CoV-2 was inactivated (≥5.5 log_10_ reduction) in cell culture, nasopharyngeal samples, and serum after 15, 10, and 10 min, respectively (Table 5). The efficacy of heat treatment against SARS-CoV-2 was also evaluated at other temperatures that varied among studies, including 70 °C [37,39,68], 92 °C [69], 95 °C [67], and 98 °C [66] (Table 5).

Overall, heat treatment at 60 °C for 60 min would lead to SARS-CoV-1 inactivation according to six studies [31,34,35,45,50,65] (~3.5–8.0 log_10_ reductions in a variety of media), and to SARS-CoV-2 inactivation as per six studies [33,37,66,67,69,70] (~4.6–7.0 log_10_ reduction) (Table 5). However, one study showed residual SARS-CoV-1 infectivity until 90 min at 65 °C [46]. In addition, Fischer et al.’s findings on dry heat treatment at 70 °C against SARS-CoV-2 are surprising and difficult to interpret, as while their figure indicated a limited ~2.2 log_10_ reduction in virus titre on stainless steel after 60 min, the decay in the control samples on the same medium at room temperature was ~1.5 log_10_ over the same period [39].

### 4.3. Chemical Disinfection

Elimination of the virus from a number of surfaces can be rapidly achieved with chemical treatment. Virucidal activity against SARS-CoV-1 and/or SARS-CoV-2 with a ≥4 log_10_ reduction has been shown for a variety of chemicals including: povidone-iodine solution (≥0.5%) [73,74], formaldehyde (4%) [33,66], ethanol (≥70%) [37,39,74,75,76], sodium hypochlorite (10% [33] or household bleach 1:49 [37]), and benzalkonium chloride (0.1%) [37]. However, liquid hydrogen peroxide at 3% had minimal viricidal effect against SARS-CoV-2 after 30 seconds of contact [74]. Chemical disinfection may be suitable for certain hard surfaces and PPE items such as goggles, but is not recommended for FFRs due to loss of filtration performance (e.g., alcohol [22,41,42,43,44]) or residual chemical odor (e.g., hypochlorite [77]).

## 5. Impact of Disinfection on FFRs

### 5.1. UVGI

Thirteen published studies and two non-peer-reviewed reports have examined the effects of UVGI on the performance and structure of FFRs (Table 6). 

Studies have assessed a range of parameters, including the FFR’s particle filtration efficiency, material strength, and “fit”. The latter quantifies how tight the seal between the respirator and the wearer’s face is, being generally derived from the ratio of non-toxic sodium chloride particles generated by the testing equipment present in the ambient air to that within the respirator on the wearer [86] (compared with a self-administered “fit check” prior to use, to determine if the FFR seals properly to the wearer’s face prior to use [87]).

Exposure methodology for UVGI varied somewhat, ranging from a single cycle to as many as 20 cycles, or UVGI exposure to the outer-facing surface of the respirators or to both surfaces (Table 6). Seven studies (with applied UVC doses ranging from 180 to 6900 mJ/cm^2^) [39,41,77,78,79,80,81] reported negligible effects on FFR filter aerosol penetration, filter airflow resistance, fit, odor detection, comfort, donning difficulty, or physical appearance (Table 6). Heimbuch and Harnish [51] evaluated the effects of multiple UVGI cycles on 15 different N95 FFR models; up to 20 UVGI cycles (total applied UVC dose 20,000 mJ/cm^2^) did not have a meaningful effect on fit, airflow resistance, or particle penetration for any model. Strap strength was unaffected by 10 UVGI cycles (total applied dose 10,000 mJ/cm^2^), but 20 cycles (20,000 mJ/cm^2^) affected the material integrity of straps in certain models [51]. In other studies, applied UVC doses of 10,000 mJ/cm^2^ had negligible effects on two N95 FFR models tested (e.g., particle filtration, polymer structure, and tensile strength) [85], and ~18,000 mJ/cm^2^ reduced fit scores of three N95 FFR models but which still remained within the required performance range [42] (Table 6).

Lindsley et al. [82] estimated the cumulative effect of extremely high exposures to UVC on N95 FFRs, in order to mimic repeated cycles of UVGI treatment. Their lowest applied dose of 120,000 mJ/cm^2^ reduced the bursting strength of the four N95 models tested by 11% to 42% (depending on the model and the individual layer), with minor effects on filter aerosol penetration and filter airflow resistance [82] (Table 6). An applied dose of 590,000 mJ/cm^2^ reduced the breaking strength of straps from the four N95 FFR models tested by 10% to 21% [82].

Of interest is the research letter by Ozog and colleagues [83], which reported marked differences in the effects of UVGI on fit testing among N95 FFR models. While one model was unaffected by a total applied UVC dose of 60,000 mJ/cm^2^, others failed fit testing after a single treatment cycle (i.e., 3000 mJ/cm^2^) (Table 6). It was concerning that some models failed fit testing even before treatment [83].

Therefore, while it seems that in general FFRs will withstand a total applied UVC dose ≥20,000 mJ/cm^2^, the evidence shows that findings from one model cannot be extrapolated to others. Further, it appears that Fischer et al. were the only researchers to investigate the combined effects of cycles of wear and UVC disinfection [39], but they have not examined more than three cycles of 2 h of wear and 1980 mJ/cm^2^ disinfection, so further studies are required looking at more repeated FFR disinfection and reuse, particularly involving extended use.

### 5.2. Heat Treatment

Table 7 summarizes 20 studies that examined the effects of heat treatment on the performance and structure of FFRs, most of which (14) were carried out in the current COVID-19 pandemic, including four that are yet to be peer reviewed. These studies consisted of dry heat treatment or moist heat treatment, with a limited number examining the effects of steam treatment (Table 7). While some studies have worked with temperatures above 100 °C (e.g., [41,77,88]), we have focused on lower temperatures, as most FFRs appear to be made of polypropylene [89], whose maximum operating temperature would be below 100 °C [90].

While moist heat has been reported to be better than dry heat at disinfection [91], the majority of studies have focused on dry heat (Table 7). This is likely because, in theory, such treatment could be relatively easily replicated, using for example, any oven with a thermostat.

The heat treatment studies were very inconsistent in regard to the adopted temperatures and length of exposure (Table 7). Thus, the findings reported from the 20 studies described in Table 7 have been further condensed in Table 8 to facilitate their interpretation. The studies that have utilised an upper temperature range of 90–100 °C (potentially damaging to plastic polymers) and found no effect on FFR fit testing or filtering performance (including two using steam treatment [44,94]) had a relatively short total cumulative treatment time ≤60 min (Table 8). In contrast, a number of studies have shown that both dry and moist heat treatment of FFRs in the range of 70–85 °C were possible for extended periods of time (Table 8), without marked effects on respirator performance and/or fit (Table 7). These have ranged from 90 to 600 min (cumulative) at 70 °C using dry or moist heat [39,68,93,96,97], to 150–400 min (cumulative) at 85 °C with low-moisture (30% RH) to 100% RH [44,92] (Table 8). Liao et al. [44] also reported that the particle filtration efficiency of the meltblown fabric of N95 FFRs was not markedly affected after 1500 min of dry heat treatment at 75 °C or after 1000 min at 85 °C and 30% RH (Table 7 and Table 8).

Of note, even at the lower end of the temperature range (60 °C), three studies from the same group using moist heat incubation (80% RH) [78,79,80] reported that while cumulative treatment times of 30–90 min did not significantly affect FFR performance and fit, there was separation of the inner foam nose cushion for a given respirator model (Table 7). In addition, as reported for UGVI treatment, some FFRs failed fit or particle filtration testing even before treatment [92,95,97], and the ability of different models to withstand high-temperature insults varied [88]. Further, while a number of studies have shown that many FFR models can withstand multiple disinfection cycles with heat at 70 to 85 °C (Table 8), the findings reported by Fisher et al. suggest that this may not be necessarily applicable in practice, when treatment cycles are interpolated with periods of actual FFR wear [39] (Table 7).

## 6. Summary of Evidence

### 6.1. Viability

The viability of both SARS-CoV-1 and SARS-CoV-2 will vary markedly depending on the material in question, the ambient temperature, the medium in which the virus is deposited, and possibly the initial viral load.

SARS-CoV-2 could potentially remain infectious for many days on inanimate objects (including PPE) under the right conditions in infectious bodily fluids, potentially as long as 3 weeks at room temperature.

### 6.2. Disinfection

We advise against attempts to disinfect and reuse soiled PPE; disinfection and viability studies show a protective effect of protein and aqueous substrata on SARS-CoV-1 and SARS-CoV-2 infectivity. Therefore, it would be ill-advised to attempt to disinfect any PPE that is clearly contaminated upon visual inspection.

The data on UVGI remain scarce, heterogeneous, conflicting, and consequently difficult to interpret. Nonetheless, the existing data indicate that an applied UVC dose of approximately 1000 mJ/cm^2^ would likely be effective against SARS-CoV-2 on a relatively flat surface and in the absence of soiling agents (e.g., bodily fluids), leading to a ~4 to 5 log_10_ TCID_50_/mL reduction in virus titre. However, based on some of the SARS-CoV-2 inactivation data from N95 FFRs [39,56], a conservative dose of 1500–2000 mJ/cm^2^ should be considered, given: (i) possible errors in applied dose estimation; (ii) uncertainties regarding the actual susceptibility of SARS-CoV-2 to UVC; (iii) the effects of different materials on SARS-CoV-2 susceptibility to UVC; and (iv) the challenge to reach the inner filtering layers of FFRs [62,98] and overcome potential shadowing effects, so that sufficient UVC is applied to their various segments (e.g., straps).

Unpublished data from our group show that there is minimal UVC radiation on the wearer-facing side of FFRs when the outer side is irradiated (outer 7.34 mW/cm^2^ vs. inner 0.10 mW/cm^2^), with 99–100% blockage of UV light in N95 FFRs also shown by Ontiveros et al. [98]. There are also reports of widespread SARS-CoV-2 infection among frontline medical staff [99], thus, it has to be assumed that SARS-CoV-2 contamination of FFRs would likely occur on both sides, particularly when there is strong evidence that asymptomatic cases may be responsible for the transmission of a large proportion of SARS-CoV-2 infections [100,101]. Therefore, we recommend that both wearer-facing and outer-facing surfaces of FFRs be equally treated at the recommended UVC dose (i.e., at a total dose 3000–4000 mJ/cm^2^).

For heat treatment, the higher the temperature, the faster the virus inactivation occurs. Conservatively, dry heat treatment at 60–65 °C for 90 min or 70–75 °C for 60 min would most likely lead to inactivation of SARS-CoV-2 on PPE, with the suggested treatment period advisable to ensure adequate heat transfer to the inner layers of FFRs, particularly if a number of respirators are being treated simultaneously (in which case we would caution against stacking them). While we cannot recommend a target RH due to the paucity of data for SARS-CoV-1 and SARS-CoV-2, there is some evidence that higher relative humidity (i.e., moist heat) would likely increase treatment efficacy [91].

### 6.3. Impact of Disinfection on FFRs

If the conservative applied UVC dose of 1500–2000 mJ/cm^2^ per FFR surface is adopted (i.e., total dose of 3000–4000 mJ/cm^2^), it may be possible to subject FFRs to approximately five UVGI disinfection and reuse cycles without compromising respirator function and material integrity. Similarly, using heat treatment at 60–65 °C for 90 min or 70–75 °C for 60 min, five disinfection cycles would likely be possible.

However, the feasibility of multiple disinfection cycles needs to be ascertained for a given FFR model, as there is extensive evidence of variability. Extended use also needs to be considered in the achievable cycle number.

Due to the widespread use of alcohol-based disinfectants, it is important to emphasise that FFRs should not be sprayed with alcohol, as it can remove the electrostatic charge from the respirator filter material, severely reducing the filter’s effectiveness at collecting particles, as shown by a number of studies [22,41,42,43,44].

## 7. Disinfection of Other PPE

While this study focused primarily on FFRs, the supply of other PPE will be seriously affected in a pandemic situation, in particular surgical masks (potentially as important as FFRs [16,17]) and isolation gowns [102], but also face shields and eye protection.

***Surgical masks***—UVGI would not be appropriate for disinfection of surgical masks due to their folded construction. Thus, heat treatment would be the most readily available option, likely at similar levels as FFRs, although 60 min at 70–75 °C would seem more appropriate to maximize thermal viricidal activity deep within mask folds.

***Isolation gowns***—Heat treatment would be recommended due to their size and folds, as wiping with chemical disinfectants would be laborious and prone to failure. We are not aware of disinfection studies undertaken on isolation gowns, but heat treatment at 60–65 °C for 90 min would be advisable as the plastic polymers that often make up isolation gowns tend to have relatively low maximum operating temperatures, and higher temperatures would likely lead to permanent structural damage.

***Goggles and other eye protection***—These should be immersed for at least 10 min in a chlorine solution at a conservative dose of 5000 mg/l, which would account for the gradual reduction in chlorine concentration throughout the day. Alternatively, in the absence of any signs of soiling, these could be thoroughly cleaned with an 80% ethanol solution for at least 30 s [75,103]. Afterwards, the goggles/eyewear should be rinsed well with warm water to remove the disinfectant solution, which could otherwise damage the equipment or cause skin irritation for the wearer. In addition, as goggles and other eyewear can be made of different materials, we recommend testing to make sure the disinfectant will not damage the equipment (e.g., ‘fogging’ the lenses) before implementing a chemical disinfection procedure.

***Face shields***—These are made of thin plastic and would likely be damaged if treated at temperatures ≥60 °C. The best approach may be to clean face shields using the same procedures as for eyewear; however, face shields usually have a foam-like material or thicker plastic band on the area that is in direct contact with the face, which may be difficult to thoroughly clean with chemical disinfectants. There is a lack of information on the use of UVGI for face shields, and they may be constructed from a wide variety of transparent plastics with different sensitivities to UVC effects. It is therefore unclear whether UVGI can be used once or repeatedly without discoloration or ‘fogging’ due to UV damage, and testing would be recommended.

## 8. Proposed Disinfection and Reuse Protocol

**Figure 1:**
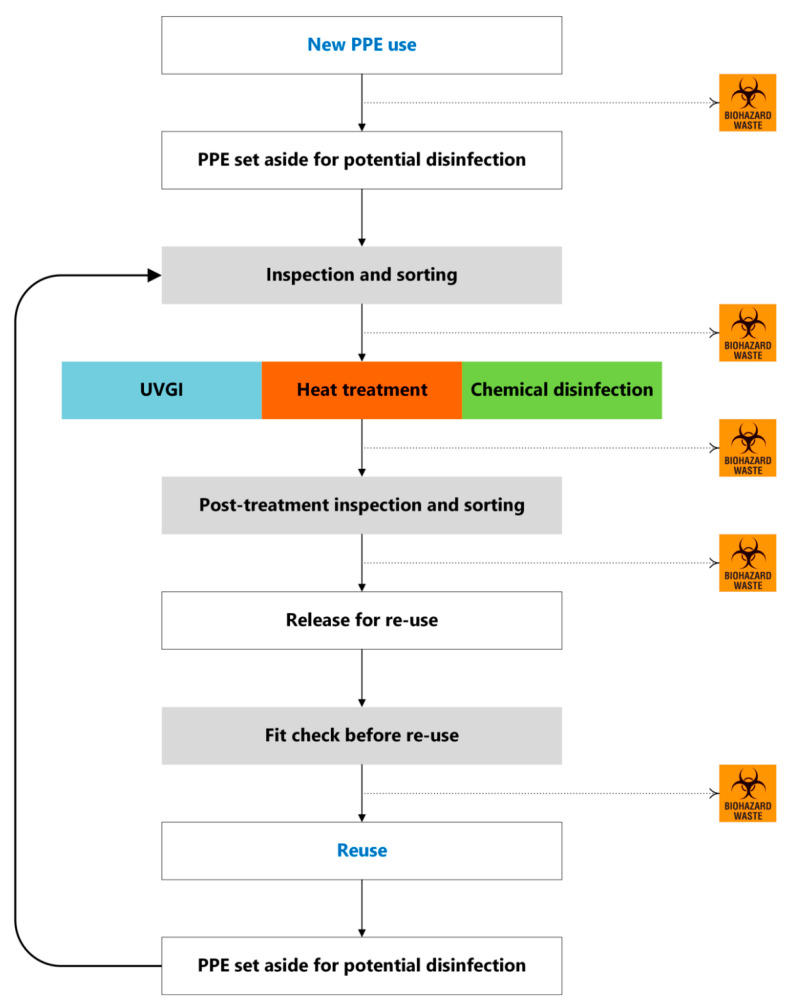
Proposed steps for a possible protocol for PPE disinfection and reuse. Dotted lines represent the path (i.e., biohazard waste) for PPE with any sign of damage or soiling.

Based on the available evidence, a possible disinfection and reuse protocol is proposed as outlined in Figure 1.

Following the use of new PPE, at point of doffing PPE, the wearer is to remove and inspect items, looking for any damage or soiling (e.g., bloodstains or presence of organic material). If the PPE is damaged or visibly contaminated, this is to be placed in a bin for biohazard waste. If not damaged or contaminated, PPE is to go into a separate clearly marked bin for reuse. This PPE is to be bagged and transported in a bin to the storage area using locally approved standard operating procedures. 

Key steps in the proposed disinfection and reuse cycle include (Figure 1):

(a) ***Inspection and sorting***—careful inspection of PPE (including straps); any soiled and damaged PPE to be discarded, intact PPE to be stored.
(b) ***Treatment***—UVGI, heat, or chemical disinfection, as appropriate.
(c) ***Re-inspection and sorting***—after disinfection, careful re-inspection of PPE (including straps of FFRs) must take place; any PPE with any sign of damage must be discarded; intact PPE to be packaged for reuse, after being appropriately marked as PPE derived from disinfection, including the number of the disinfection cycle.
(d) ***Fit checking***—frontline staff to ensure that FFR passes fit check prior to use, and any disinfected PPE fit properly. At any sign of suboptimal fit, disinfected PPE to be immediately discarded.

It is likely that FFRs should be discarded after the fifth reuse, although further research is required to determine the number of cycles possible. An exception to this rule would be under extreme circumstances, where the alternative to further reuse of suboptimal PPE would be not wearing any protection at all.

This protocol provides recommendations for a possible pragmatic disinfection process for most PPE, that could be rapidly implemented, based on best available evidence. 

## 9. Cautionary Notes

### 9.1. Reuse of FFRs

In an ideal world, the reuse of FFRs is not encouraged if at all possible, as complete disinfection cannot be guaranteed for all FFRs under all circumstances. As highlighted by the US Centers for Disease Control and Protection (CDC), it is not possible to determine a maximum possible generic number of safe reuses for FFRs [104]. Nonetheless, the CDC recommend that in the absence of the manufacturer’s guidance, FFRs should not be reused more than five times [104], as suggested by two previous studies [89,105] based on the observed reduction in FFR fit.

Importantly, at least one small study showed that, unsurprisingly, the reuse of N95 FFRs was associated with higher fit failure rate [106]. Thus, it is fundamental that the combination of disinfection and reuse are properly investigated, as the number of paired cycles that FFRs can be subjected to would most likely be lower than the number of disinfection cycles alone.

### 9.2. Extended Use of FFRs

According to Fisher and Shaffer 2014 [89], extended use would be preferable over limited reuse due to a lower risk of contamination with lesser contact with FFR surface. However, extended used leads to an increase in non-adherent behaviors (e.g., adjusting or touching the FFR) over time [107], increasing the risk of self-contamination. In a recent study from China during the COVID-19 response, 97% of 542 frontline health care workers had some form of skin damage, which was greater with longer wear of FFRs [108]. An accompanying editorial highlighted that this increases the likelihood of non-adherent FFR-wearing behavior, and consequently an increased risk of viral transmission [109]. Notably, cases of contact dermatitis in health care workers are relatively common [108,110], including those resulting from the use of FFRs [108,111,112,113,114,115,116]. Thus, it is almost inevitable that the prevalence of dermatological conditions would increase with extended use of PPE. As prolonged skin breakdown increases health care workers susceptibility to infection and improper PPE use, access to virtual dermatology clinics for health care workers is strongly recommended to manage and treat skin breakdown in health professionals wearing PPE for extended periods. Further, it is also important to attempt to mitigate other adverse effects associated with extended use of FFRs, such as headaches [117].

### 9.3. Ultraviolet Light Toxicity

While UVC is perfectly safe when the equipment in question is appropriately designed and handled, it is worth stressing that accidental exposure to UVC is harmful to humans [118]. There has been at least one case in the current pandemic scenario of photokeratitis and epidermal phototoxicity caused by the improper use of a UVC disinfection apparatus in the home environment [119]. Therefore, we advise against the use of homemade devices for PPE disinfection using UVGI.

### 9.4. Other Pathogens

While different regimens of UVGI and heat treatment are effective against a large number of human pathogens, there is a high degree of variability among the susceptibility to temperature and UVC among microorganisms [59,120]. Thus, while the disinfection procedures reviewed here have focused on SARS-CoV-1 and SARS-CoV-2, they would not necessarily be efficacious against all other pathogens, in particular spore-forming bacteria. However, we contend they would likely be effective against many important human pathogens, and Xiang et al. for example [96] showed that dry heat treatment of FFRs at 60 °C for at least 60 min inactivated H1N1 virus, one fungus (*Candida albicans*), and six bacterial species (including *Escherichia coli*, *Staphylococcus aureus*, and *Pseudomonas aeruginosa*). The literature on UVGI is extensive, and the applied doses proposed here would be effective against a large number of human pathogens [59,98], but covering these would be outside the scope of this review. Nonetheless, the potential risk posed by other pathogens is another reason to make sure that visibly soiled PPE is not reused, as disinfection would be more difficult to achieve.

## 10. Conclusions

Given the shortage of equipment for frontline staff worldwide, the authors believe that there is sufficient evidence to support the disinfection and potential reuse of FFRs and other PPE in the current pandemic scenario when necessary. The authors have applied a would-I-wear-it (WIWI) test to the process for developing protocol recommendations. Further, based on the literature that was examined, the proposed methodology would likely achieve disinfection against most other important pathogenic organisms.

Proper disinfection and reuse of PPE would not only address the problem of short-term supply in the frontline during the pandemic, but also likely lead to considerable cost savings in the long term. Further, it would also improve the environmental footprint of a given health care facility, potentially allowing for consideration of long-term reuse of PPE. According to estimates from US hospitals for example, 5.17 tons of waste are generated per staffed bed every year [121], and in the current COVID-19 pandemic, increases of as much as 280 tons/day of extra medical waste have been reported in Southeast Asia [122]. Ultimately, it is the right of every health care worker responding to the current pandemic to have PPE available not only for their protection, but also to reduce the spread of COVID-19 [123].

## Figures and Tables

**Table 1:** Databases/search engines used for this review, including combinations of keywords and Boolean operators, and number of results yielded as of 18 July 2020.

Database	Results (*n*)	Search Terms
PubMed	1439	((“2003/01/01”[Date-Publication]: “3000”[Date-Publication]) AND (SARS[Title/Abstract]) AND ((steril* [Title/Abstract]) OR (surviv* [Title/Abstract]) OR (viability[Title/Abstract]) OR (N95[Title/Abstract]) OR (PPE[Title/Abstract]) OR (“personal protect*”[Title/Abstract]) OR (disinfect* [Title/Abstract]) OR (decontaminat* [Title/Abstract]) OR (inactivat* [Title/Abstract]) OR (heat[Title/Abstract]) OR (ultraviolet[Title/Abstract]) OR (UV[Title/Abstract]))
Web of Science ^‡^	1468	((TI = SARS) OR (AB = SARS)) AND (TI = (ultraviolet OR UV OR heat OR N95 OR PPE OR “personal protect*” OR surviv* OR viability OR disinfect* OR decontam* OR inactivat*)) OR (AB = (ultraviolet OR UV OR heat OR N95 OR PPE OR “personal protect*” OR surviv* OR disinfect* OR decontam* OR inactivat* OR viability))
Google Scholar ^†^	~182,000	SARS AND (ultraviolet OR UV OR heat OR inactivation OR inactivate OR decontaminate OR decontamination OR disinfect OR disinfection OR N95 OR PPE OR “personal protective” OR “personal protection” OR survival OR survivorship OR viability)

^‡^ Search included three databases: Web of Science Core Collection, Current Contents Connect, and SciELO Citation Index. ^†^ Search excluded patents; results were sorted automatically by relevance based on the search engine’s own ranking algorithms, and the top 2500 results were screened.

**Table 2:** Studies reporting on the viability of SARS-CoV-1.

Study	Inoculum and Conditions	Materials and Time to Inactivation
Duan 2003 [28]	6 log_10_ TCID_50_ in 300 μL Room temperature (~20 °C) LOD not reported	Wood board, mosaic—4 days Glass, press paper, plastic, water, soil—5 days Metal, cloth, filter paper—virus still detected after 5 days Serum, filtrated sputum—4 days Sputum, faeces, filtrated faeces, urine—virus still detected after 5 days
Bao 2003 [35]	~6.0–7.5 log_10_ TCID_50_ 3 temperatures: 4 °C, room temperature (24.5 °C), and 37 °C	4 °C—~4.0 log_10_ reduction after 15 days, but no data thereafter Room temperature—~3.5 log_10_ TCID_50_ reduction after 5 days, but no data thereafter 37 °C—72 h (~6.0 log_10_ reduction)
Li 2003 [29]	Initially 3.0–6.0 log_10_ TCID_50_ Inactivation likely <1.0 log_10_ TCID_50_	Cloth—6 h (≥4.0 log_10_ reduction) Soil—24 h (≥5.0 log_10_ reduction) Filter paper—48 h (≥3.5 log_10_ reduction) Wood—48 h (≥2.0 log_10_ reduction) Stainless steel and glass—48 h (≥5 log_10_ reduction) Plastic—48 h (≥ 4.7 log_10_ reduction)
Lai 2005 [30]	6.0–6.8 log_10_ TCID_50_/mL	Cotton gown, paper—24 h (6.0 log_10_ reduction) Disposable polypropylene gown—2 days (6.0 log_10_ reduction) Nasopharyngeal aspirate or throat and nasal swab at room temperature—10 days (~6.0 log_10_ reduction) Nasopharyngeal aspirate at 4 °C— ~3.8 log_10_ reduction after 27 days, but no data thereafter
Rabenau 2005 [31]	500 μL virus suspension (~6.7 log_10_ TCID_50_/mL) applied to dish and left to dry at 21–25 °C, unknown RH LOD = ~1.7 log_10_ TCID_50_/mL	Plastic (polystyrene petri dish)—infectivity only lost after 9 days (~5.0 log_10_ reduction) In suspension (10% FBS) remained infective after 9 days (just ~1.2 log_10_ reduction), with no data thereafter
Pagat 2007 [34]	22 ± 3 °C, 10–25% RH Virus solution (~7.7 log_10_ TCID_50_/mL) left to dry on glass petri dish LOD = 1.5 log_10_ TCID_50_/mL	Taken 42 days until below LOD (~6.2 log_10_ reduction) Authors noted that surface decay was much faster in solution than dried samples
Chan 2011 [32]	7 log_10_ TCID_50_/mL 22–25 °C, 40–50% RH	Plastic well plate—infectivity lost after 21 days in dried form (i.e., 7 log_10_ reduction) and 28 days in solution.
Chan 2020 [33]	7 log_10_ TCID_50_/mL 10 μL droplets of virus culture on a glass slide Inactivation ≈6 log_10_ reduction	4 °C—only 2 log reduction after 14 days in dried form; <1 log_10_ loss in solution, but no data thereafter 20–25 °C (RH 63%)—14 days in both forms 33 °C – 3 days in dried form; 5 days in solution 37 °C—3 days in both forms
van Doremalen 2020 [27]	3.4–3.7 log_10_ TCID_50_/mL 21–23 °C, 40% RH Surface deposits of 50 μL	Cardboard—24 h (~2.1 log_10_ reduction) Copper—24 h (~1.9 log_10_ reduction) Stainless steel—3 days (3.1 log_10_ reduction) Plastic—4 days (2.9 log_10_ reduction)

BSA, bovine serum albumin; HL, half-life; FBS, fetal bovine serum; LOD, limit of detection; RH, relative humidity; TCID_50_, median tissue culture infectious dose, corresponding to the concentration at which 50% of the experimental cells are infected after inoculation.

**Table 3:** Studies reporting on the viability of SARS-CoV-2.

Study	Inoculum and Conditions	Materials and Time to Inactivation
Behzadinasab 2020 [36]	~5.8–6.1 log_10_ TCID_50_/mL, 5 μL droplets 22–23 °C, 60–70% RH	Glass—~2.3 log_10_ reduction after 24 h, but no data thereafter Stainless steel—~1.8 log_10_ reduction after 24 h, but no data thereafter
Biryukov 2020 [38]	~2 log_10_ TCID_50_/mL 5 μL droplets LOD = 0.2 log_10_ TCID_50_/mL Inactivation ≈1.8 log_10_ reduction	Stainless steel—inactivation after 24 h at 35 °C and 20%, 40%, and 60% RH; after 48 h at 24 °C and 40% and 60% RH, but not at 20% RH (no data thereafter) Results reportedly similar in acrylonitrile butadiene styrene plastic and nitrile rubber
Chan 2020 [33]	6.5 log_10_ TCID_50_/mL 10 μL droplets of virus culture on a glass slide Inactivation ≈5 log reduction	4 °C—only 2 log_10_ reduction after 14 days in dried form or solution, but no data thereafter 20–25 °C (RH 63%)—5 days in dried form and 14 days in solution 33 °C—3 days in dried both forms 37 °C—24 h in dried form and 3 days in solution
Chin 2020 [37]	Temperature decay: 6.5 log_10_ TCID_50_/mL LOD = 2 log_10_ TCID_50_/mL Surface decay at room temperature (22 °C, 65% RH): 4.8–6.1 log_10_ TCID_50_/mL 5 μL droplets of virus culture LOD = 2 log_10_ TCID_50_/mL	Temperature decay: 4 °C—only 0.7 log_10_ reduction after 14 days 22 °C—14 days (≥4.5 log_10_ reduction) 37 °C—2 days (≥4.5 log_10_ reduction) Room temperature study: Printing paper—3 h (≥2.8 log_10_ reduction) Tissue paper—3 h (≥3.5 log_10_ reduction) Cloth—2 days (≥2.8 log_10_ reduction) Wood—2 days (≥3.7 log_10_ reduction) Glass—4 days (≥3.8 log_10_ reduction) Banknote—4 days (≥4.0 log_10_ reduction) Plastic—7 days (≥4.8 log_10_ reduction) Stainless steel—7 days (≥4.8 log_10_ reduction) Surgical mask inner layer—7 days (≥4.8 log_10_ reduction) Surgical mask outer layer—3.0 log_10_ reduction after 7 days, but there was remaining infectivity (no data thereafter)
Fischer 2020 [39]	~4.5 log_10_ TCID_50_/mL 50 μL inoculum on stainless steel and N95 disks 21–23 °C, 40% RH LOD = 0.5 log_10_ TCID_50_/mL	N95 respirator—24 h (≥4 log_10_ reduction) Stainless steel—48 h (≥4 log_10_ reduction)
Kasloff 2020 [40] (!)	1–1.4 cm^2^ coupons 10 μL droplets ~5.8 log_10_ TCID_50_/mL inoculum in soil load (mucin + BSA + tryptone) ~20 °C, 35–40% RH LOD = 0.8 log_10_ TCID_50_/mL (inactivation ≈5.0 log_10_ reduction)	100% cotton t-shirt fabric—1 day Chemical-resistant nitrile rubber gloves—7 days Nitrile rubber gloves—14 days Stainless steel, plastic face shield, N100 respirator, and polyethylene coveralls—21 days N95 respirator—~4.9 log_10_ reduction after 21 days, but LOD not reached (no data thereafter)
van Doremalen 2020 [27]	~3.2–3.7 log_10_ TCID_50_/mL 21–23 °C, 40% RH Surface deposits of 50 μL	Plastic and stainless steel—4 days (3.2 log_10_ reduction) Cardboard—2 days (~2.0 log_10_ reduction) Copper—8 h (~1.7 log_10_ reduction)

BSA, bovine serum albumin; LOD, limit of detection; RH, relative humidity; TCID_50_, median tissue culture infectious dose, corresponding to the concentration at which 50% of the experimental cells are infected after inoculation. (!) Study only available as preprint at the time of manuscript preparation and therefore yet to be peer reviewed.

**Table 4:** Studies reporting on the efficacy of ultraviolet germicidal irradiation (UVGI) against SARS-CoV-1 and SARS-CoV-2.

Virus	Study	Inoculum and Conditions	UV Exposure	Key Findings and Notes
SARS-CoV-1	Duan 2003 [28]	6 log_10_ TCID_50_ in 100 μL culture medium in well plates	260 nm-length UVC Irradiance: >90 μW/cm^2^ Distance: 80 cm	Cell culture exposure—undetectable CPE with 300 mJ/cm^2^
	Ansaldi 2004 [49]	“standard concentration of cell-grown virus” 1 mL salt solution on a cell culture plate 18 °C, 40% RH	Irradiance: 40 mW/cm^2^ UV type and distance to light undisclosed	Negative result by cell culture and PCR with 12,000 mJ/cm^2^ Methods not fully described
	Darnell 2004 [46]	2 mL aliquots of virus in well plates ~5.7 log_10_ TCID_50_/mL LOD = 1.0 log_10_ TCID_50_/mL	UVC 254 nm Irradiance: 4016 μW/cm^2^ at 3 cm	~4.6 log_10_ reduction with ~1450 mJ/cm^2^ Complete inactivation (≥4.7 log_10_ reduction) achieved with 3600 mJ/cm^2^ UVA (365 nm) completely ineffective at a total applied dose of 1920 mJ/cm^2^
	Darnell 2006 [45]	Virus solution in well plates ~5.0 log_10_ TCID_50_/mL LOD = 1.0 log_10_ TCID_50_/mL	UVC 254 nm Irradiance: 4016 μW/cm^2^ at 3 cm	Study specific to non-cellular blood products Inactivation in PBS solution (≥4.0 log_10_ reduction) with 9600 mJ/cm^2^ Incomplete inactivation in BSA protein solutions with 14,500 mJ/cm^2^
	Kariwa 2006 [50]	2 mL aliquots open plastic petri dishes 7.6 log_10_ TCID_50_/mL LOD = 1.0 log_10_ TCID_50_/mL	UV “normal biosafety cabinet UV lights” (likely UVC) Irradiance: 134 μW/cm^2^	~5.3 log_10_ reduction with 121 mJ/cm^2^, but LOD not reached with 500 mJ/cm^2^ (maximum applied dose tested)
	Heimbuch 2019 [51]	FFR coupons in 3 soiled conditions: no soiling agent artificial saliva (mucin) artificial skin oil (sebum) Controls at 4.6–5.5 log_10_ TCID_50_/mL	UVC lamp (254 nm) Distance 15.2–22.9 cm Irradiance: mean 2.3 mW/cm^2^	No detectable viable virus in the 3 conditions tested with 1000 mJ/cm^2^ (i.e., ≥4.0 log_10_ reduction)
	Eickmann 2020 [52]	375 mL platelet concentrates 5.9 log_10_ TCID_50_/mL	THERAFLEX UV-Platelets system (UVC 254 nm) up to 200 mJ/cm^2^	Below LOD (i.e., ≥3.4 log_10_ reduction) with 100 mJ/cm^2^ Note that the system employed achieves virus inactivation more efficiently through vigorous agitation of fluid bags [53]
SARS-CoV-2	Fischer 2020 [39]	~4.5 log_10_ TCID_50_/mL 50 μL inoculum on stainless steel and N95 disks LOD = 0.5 log_10_ TCID_50_/mL	UVC 260-285 nm Irradiance 0.55 mW/cm^2^ at point of exposure	Stainless steel—below LOD (≥4 log_10_ reduction) with 330 mJ/cm^2^ N95—LOD not reached with 1980 mJ/cm^2^ (visually estimated from figure as ~3 log_10_ reduction), but no data thereafter (i.e., beyond 60 min)
	Heilingloh 2020 [54]	600 µL 6.7 log_10_ TCID_50_/mL in 24 well plates	UVC 254 nm at 1.94 mW/cm^2^ UVA 365 nm at 0.54 mW/cm^2^	UVC achieved >6.7 log_10_ reduction at 1048 mJ/cm^2^ UVA achieved 1.0 log_10_ reduction at 292 mJ/cm^2^ applied dose
	Inagaki 2020 [55]	150 μL with 4.3 log_10_ plaque forming units (PFU)/mL in 60 mm petri dish LOD = ~1.3 log_10_ PFU/mL	Deep ultraviolet light-emitting diode (DUV-LED) 280 ±5 nm Irradiance 3.75 mW/cm^2^ at 20 mm	Below LOD (~3.2 log_10_ PFU/mL reduction) with 75 mJ/cm^2^
	Smith 2020 [42]	100 μL of saline/albumin solution with high viral titer “directly infiltrated” into strips from 3 different N95 models, aiming to ‘expose’ the middle layer	UVC 254 nm Applied dose 630 mJ/cm^2^ to each side (i.e., 1260 mJ/cm^2^ per sample)	UVC did not inactivate the virus from the N95 samples Authors commented that it would be “hard to imagine a realistic scenario where healthcare workers would face this degree of mask inoculum”
	Ozog 2020 [56](!)	10 μL droplet viral stock (≤6.0 log_10_ TCID_50_/mL) LOD ≈ 1.8 log_10_ TCID_50_/mL, so inactivation up to ~4.2 log_10_ reduction	UVC 254 nm UVGI device with 4 lamps, irradiance of 16 mW/cm^2^ at 11.5 cm away Single applied dosed of 1500 mJ/cm^2^ tested	Four N95 FFR models tested, each with 4 locations tested (nosepiece, apex, chin-piece, and strap), with 3 samples each Most facepiece samples (total n = 32) had viral loads <LOD, but 4 samples from 2 models did not For straps, all samples from 2 models (3 each) were <LOD, but only 1/6 samples were <LOD for other 2 models
	Ratnesar-Shumate 2020 [57]	5 μL droplets of viral suspension (‘simulated’ saliva’ or FBS) on stainless steel coupons Virus concentration unspecified but estimated from graphs	UVB 280–315 nm Irradiance 0.16 mW/cm^2^ for up to 20 min (maximum applied dose 192 mJ/cm^2^)	‘Simulated’ saliva: ~2.5 log_10_ reduction (from ~3 to ~0.5 log_10_ TCID_50_/mL) FBS: ~1.1 log_10_ reduction (from ~2.6 to ~1.5 log_10_ TCID_50_/mL) Study aimed to demonstrate SARS-CoV-2 inactivation by sunlight (which does not include the UVC spectrum)

BSA, bovine serum albumin; CPE, cytopathic effect; FBS, fetal bovine serum; FFR, filtering facepiece respirator; LOD, limit of detection; PBS, phosphate-buffered saline; PCR, polymerase chain reaction; RH, relative humidity; TCID_50_, median tissue culture infectious dose, corresponding to the concentration at which 50% of the experimental cells are infected after inoculation; UVA, ultraviolet light A; and UVC, ultraviolet light C. (!) Study only available as preprint at the time of manuscript preparation and therefore yet to be peer reviewed. Where necessary, the applied dose of ultraviolet light (in mJ/cm^2^) was calculated by the authors using the standard formula, as the product of irradiance (mW/cm^2^) and time (seconds).

**Table 5:** Studies reporting on the efficacy of heat treatment against SARS-CoV-1 and SARS-CoV-2.

Virus	Study	Inoculum and Conditions	Heat Treatment Details and Time to Inactivation
SARS-CoV-1	Bao 2003 [35]	Initial virus titre 8.0 log_10_ TCID_50_ RH not reported	56 °C—30 min (~8.0 log_10_ reduction) 70 °C—15 min (~8.0 log_10_ reduction) However, results showed: 6.5 log_10_ reduction after 10 min at 56 °C 7.0 log_10_ reduction after 5 min at 70 °C
	Duan 2003 [28]	6 log_10_ TCID_50_ in 100 μL culture medium in well plates LOD and RH not reported	Inactivation likely ≥4.0 log_10_ TCID_50_ 56 °C—90 min 67 °C—60 min 75 °C—30 min
	Darnell 2004 [46]	320 μL in 1.5 mL polypropylene cryotubes 5–6 log_10_ TCID_50_/mL LOD = 1.0 log_10_ TCID_50_/mL RH undisclosed	56 °C—~4.5–5.0 log_10_ reduction by 20 min, but residual infectivity remained until 90 min (≥5.0 log_10_ reduction) 65 °C—~4.0 log_10_ reduction by 5 min, but some infectivity remained until 90 min (≥4.5 log_10_ reduction) 75 °C—45 min (≥4.3 log_10_ reduction)
	Yunoki 2004 [65]	4.5 to 7.0 log_10_ TCID_50_/mL 4 different plasma products spiked with virus Heat treatment “in liquid” (not fully explained)	Virus <LOD after 30 min at 60 °C in: heat-treated/polyethylene glycol-treated intravenous immunoglobulin preparation (~3.0 log_10_ reduction), haptoglobin preparation (~5.5 log_10_ reduction), and 25% human serum albumin preparation (~4.5 log_10_ reduction) Virus in an antithrombin III preparation only inactivated after 60 min at 60 °C (~4.2 log_10_ reduction)
	Rabenau 2005 [31]	500 μL solutions with virus 7.2 log_10_ TCID_50_/mL LOD ≈ 1.8 log_10_ TCID_50_/mL Unknown RH	56 °C—30 min (≥5.0 log_10_ reduction), but this did not happen in presence of protein additive (20% FBS) with ~1.9 log_10_ reduction after 30 min (no data thereafter) 60 °C—30 min (≥5.0 log_10_ reduction), regardless of protein additive
	Darnell 2006 [45]	Samples incubated in heated water bath 4.2–5.2 log_10_ TCID_50_/mL LOD = 1.0 log_10_ TCID_50_/mL Undisclosed RH	Study specific to non-cellular blood products Human serum—56 °C for 20 min/65 °C for 10 min (~4.2 log_10_ reduction) Protein solutions—60 °C for 30 min (at highest protein content) (≥3.5 log_10_ reduction)
	Kariwa 2006 [50]	Aliquots of virus solution placed in 50 mL tubes Heated in 56 °C water bath 7.4 log_10_ TCID_50_/mL	~5.8 log_10_ reduction after 5 min at 56 °C Residual activity remained, with complete inactivation after 60 min (≥6.4 log_10_ reduction)
	Pagat 2007 [34]	Virus solution (~6.5 log_10_ TCID_50_/mL) Heated in water bath LOD = 1.5 log_10_ TCID_50_/mL	58 °C—60 min (≥5.0 log_10_ reduction) 68 °C—30 min (≥4.7 log_10_ reduction)
SARS-CoV-2	Auerswald 2020 [66](!)	140 μL aliquots of the virus (~5.8 log_10_ TCID_50_/mL) solution in well plates	56 °C—30 min (≥5.0 log_10_ reduction) 98 °C—2 min (≥5.0 log_10_ reduction)
	Batéjat 2020 [67](!)	~6.2–6.7 log_10_ TCID_50_/mL in 3 media: cell culture, nasopharyngeal samples, and serum LOD = 0.7 log_10_ TCID_50_/mL	Cell culture—after 30 min at 56 °C and 15 min at 65 °C (≥5.9 log_10_ reduction) Nasopharyngeal samples—after 10 min at 65 °C and 3 min at 95 °C (≥5.9 log_10_ reduction) Serum—after 15 min at 65 °C (≥5.5 log_10_ reduction)
	Chan 2020 [33]	30 μL of virus at 5.5 log_10_ TCID_50_/mL + 270 μL of FBS	3 log_10_ reduction in virus viability after 30 min at 56 °C Complete inactivation not achieved, but apparently not specifically aimed for
	Chin 2020 [37]	5.3–6.7 log_10_ TCID_50_/mL in cell culture medium (volume of solution not reported) LOD = 2.0 log_10_ TCID_50_/mL	56 °C—30 min (≥4.6 log_10_ reduction) 70 °C—5 min (≥3.3 log_10_ reduction)
	Daeschler 2020 [68]	5 μL of virus inoculum at 7.8 log_10_ TCID_50_/mL on 1 cm^2^ coupons from N95 respirators Control samples: 5.2–5.8 log_10_ TCID_50_/mL LOD = 2.0 log_10_ TCID_50_/mL	60 min at 70 °C and 0% RH (3.2–3.8 log_10_ reduction) Effect unchanged with and without a 5 min cool-down period mid cycle
	Fischer 2020 [39]	~4.5 log_10_ TCID_50_/mL 50 μL inoculum on stainless steel and N95 disks LOD = 0.5 log_10_ TCID_50_/mL Dry oven, RH not reported	N95—60 min at 70 °C (≥3.5 log_10_ reduction) Stainless steel—only 2.0 log_10_ reduction after 60 min at 70 °C (no data thereafter)
	Pastorino 2020 [69]	5 to 6 log_10_ TCID_50_/mL in 300 μL aliquots of 3 media: cell supernatant, human nasopharyngeal sample, and human blood serum Dry oven, RH not reported LOD = 0.5 log_10_ TCID_50_/mL	Nasopharyngeal sample, blood sera—30 min at 56 °C and 60 min at 60 °C (≥5.0 log_10_ reduction) Cell supernatant—some samples with inactivation incomplete after 30 min at 56 °C without BSA and after 60 min at 60 °C with BSA (>5.0 log_10_ but <6.0 log_10_ reduction). Below LOD after 15 min at 92 °C (≥6.0 log_10_ reduction).
	Wang 2020 [70](!)	Unreported volume of virus stocks 7.2 log_10_ TCID_50_/mL Heating conditions or RH undisclosed	37 °C—after 48 h 6.0 log_10_ reduction but some infectivity remained (no data thereafter) 42 °C—after 24 h 6.0 log_10_ reduction but some infectivity remained, which disappeared after 48 h 56 °C—30 min (7.0 log_10_ reduction) 60 °C—15 min (7.0 log_10_ reduction)

BSA, bovine serum albumin; FBS, fetal bovine serum; LOD, limit of detection; RH, relative humidity; TCID_50_, median tissue culture infectious dose, corresponding to the concentration at which 50% of the experimental cells are infected after inoculation. (!) Studies only available as preprint at the time of manuscript preparation and therefore yet to be peer reviewed.

**Table 6:** Studies reporting on the effects of ultraviolet germicidal irradiation (UVGI) on filtering facepiece respirators (FFRs).

Study	Treatment Details	FFRs	Key Findings
Viscusi 2007 [41]	Laminar flow cabinet with a 40 W UVC light (254 nm) Irradiance of 0.24 mW/cm^2^ Treatment 1: 30 min, total applied dose 400 mJ/cm^2^ [200 mJ/cm^2^ per side (i.e., inner and outer)] Treatment 2: 8 hr, total applied dose 6900 mJ/cm^2^ (3450 mJ/cm^2^ per side)	1 unidentified N95 FFR model	Average filter particle penetration not significantly affected by either treatment. No “significant visible changes” observed for any samples after either treatment.
Viscusi 2009 [77]	Laminar flow cabinet with a 40 W UVC light (254 nm) Average irradiance 0.18 to 0.20 mW/cm^2^ 15 min exposure to each side (outer and inner) Total applied dose ~180 mJ/cm^2^ per side	Not identified by the authors, but included 3 N95 FFRs and 3 surgical N95 respirators	No effect on filter aerosol penetration, filter airflow resistance, or physical appearance.
Bergman 2010 [78]	UVC lamp 40 W (254 nm) 45 min exposure at 1.8 mW/cm^2^ (total applied dose 4900 mJ/cm^2^) Distance ~25 cm Only the exteriors of the FFRs were exposed	Authors reported using the same equipment as in Viscusi 2009 [77], i.e., 3 N95 FFRs and 3 surgical N95 respirators	UVGI-treated samples had required levels of filter aerosol penetration and filter airflow resistance. UVGI-treated samples had similar mean % penetration to the treated samples tested in Viscusi 2009 [77] at much lower applied doses. There was no observed physical damage to the FFRs.
Bergman 2011 [79]	Laminar flow cabinet with a 40 W UVC lamp (254 nm) Irradiance of 1.8 mW/cm^2^ 15 min exposure to outer FFR side (total applied dose 1600 mJ/cm^2^)	3M 1860, 3M 1870, and Kimberly Clark PFR95-270	There were no significant changes in FFR fit. There was no observed physical damage to the FFRs.
Viscusi 2011 [80]	Laminar flow cabinet with a 40 W UVC lamp (254 nm) Irradiance of 1.8 mW/cm^2^ Total exposure 30 min (15 min inner side and 15 min outer side) Applied dose 1600 mJ/cm^2^ per side	3M 8000, 3M 8210, Moldex 2200, 3M 1860, 3M 1870, and Kimberly Clark PFR95-270	Authors concluded that UVGI unlikely to lead to significant changes in fit, odor detection, comfort, or donning difficulty. One subject stated that a UVGI-treated Moldex 2200 had an intolerable odor afterwards.
Lore 2012 [81]	Laminar flow cabinet, with dual-bulb 15 W UVC lamp (254 nm), 25 cm above surface Irradiance 1.6 to 2.2 mW/cm^2^ Maximum total applied dose ~1980 mJ/cm^2^ over 15 min	3M 1860s, 3M 1870	There was no significant decrease in filter performance.
Lindsley 2015 [82]	UVC (254 nm) 91 × 31 × 64 cm chamber ~27 °C at 25% relative humidity Respirator coupons: 0, 120, 240, 470, 710, or 950 J/cm^2^ of UVC on each side (one side was exposed at a time) Respirator straps: 0, 590, 1180, or 2360 J/cm^2^	3M 1860, 3M 9210, Gerson 1730, and Kimberly-Clark 46727	Slight decrease in particle penetration, estimated as up to ~1 percentage point. Small increase in flow resistance (<6% of the original value), independent of applied UV dose. At ≥710 J/cm^2^ there was major loss of bursting strength for most respirator layers tested, some as much as 90%. For some layers of certain models (3M 9210 and K-C 46727) loss >80% occurred at 470 J/cm^2^. At 590 J/cm^2^ the mean strap breaking strengths decreased by 10–21%. The lowest applied dose tested of 120 J/cm^2^ reduced the bursting strength of the four models tested by 11% to 42% (depending on layer and model).
Heimbuch 2019 [51]	UVC (254 nm) 10 or 20 cycles of 1000 mJ/cm^2^, i.e., total applied doses of 10,000 or 20,000 mJ/cm^2^ per FFR, respectively	15 models tested 10 cycles–3M 1860, 3M 1870, 3M VFlex 1805, Alpha Protech 695, Gerson 1730, Kimberly-Clark PFR, Moldex 1512, Moldex 1712, Moldex EZ-22, Precept 65-3395, Prestige Ameritech RP88020, Sperian HC-NB095, Sperian HC-NB295, US Safety AD2N95A, and US Safety AD4N95 20 cycles–3M 1860, 3M 1870, 3M VFlex 1805, Kimberly-Clark PFR, Moldex 151 2, and US Safety AD4N95	Up to 20 cycles of UVGI treatment (20,000 mJ/cm^2^) did not have a meaningful effect on fit, airflow resistance, or particle penetration for any model tested. Strap strength was unaffected by 10 UVGI cycles, but 20 cycles had some effect on certain models.
Fischer 2020 [39]	UVC LED lamp (160–285 nm) Up to 3 cycles of 2 h of wear and likely 60 min of UV treatment (estimated applied dose to flat disks 50 cm from light was 1980 mJ/cm^2^ per 60 min cycle)	3M 9211+	Study difficult to interpret as aspects of UV disinfection were insufficiently reported. Negligible effect on filtration performance after 2 cycles (~3960 mJ/cm^2^), but more marked after 3 cycles, although still within acceptable range.
Liao 2020 [44]	Sterilizer cabinet 8 W bulb UVC (254 nm) Irradiance not described 10 cycles of 30 min	15 × 15 cm pieces of meltblown fabric, described as most important N95 FFR layer	The ten 30 min cycles did not affect the fabric’s filtration efficiency. In the absence of information on irradiance, it is not possible to ascertain the actual applied UVC dose.
Ou 2020 [43]	UVC (200–280 nm) and UVB (280–315 nm) Actual applied dose reaching FFRs unknown; delivered with Xenex LightStrike Germ-Zapping Robots for 5 min within <1 m—authors estimated as >1000 mJ/cm^2^	3M 8210	There were negligible effects on particle filtration efficiency after 10 cycles (which the authors would have estimated as a total applied UVC dose >10,000 mJ/cm^2^).
Ozog 2020 [83] (!)	UVC (254 nm) Each cycle consisted of 1500 mJ/cm^2^ to the outside-facing surface plus 1500 mJ/cm^2^ to the wearer-facing surface.	3M 1860, 3M 8210, 3M 9210, Moldex 1512, and Cardinal Health N95 R/S Respirator	Only fit testing assessed; FFRs had the following number of cycles passed and cumulative UVC doses: 3M 1860—20 cycles, 60,000 mJ/cm^2^; 3M 9210 and Moldex 1512—2 cycles, 6000 mJ/cm^2^; 3M 8210 and Cardinal Health—1 cycle, 3000 mJ/cm^2^. Note that other N95 models failed fit testing before treatment
Price 2020 [84] (!)	Ten 30 min cycles of UVC (254 nm) in a sterilizer cabinet equipped with 8 W UV light bulb, interspaced with 10 min stand-down periods No quantified information on irradiance or approximate dose	3M 8200, 3M 8511, 4C AIR KN95, and Jackson R20	After 10 cycles of UVGI, there was material failure of one model and fit factor reductions of 35% to 96% depending on model. While all models failed after 10 cycles, it is not possible to interpret their results due to complete absence of data on applied dose. However, the respirators were exposed to 5 h of UVC treatment likely equating to very large doses.
Smith 2020 [42]	UVC (254 nm) 18,400 mJ/cm^2^ to exterior surface and 4600 mJ/cm^2^ to interior surface of N95 respirators	3M 1860, 3M 1870+, and 3M 8511	There was a reduction in fit scores after UVC treatment across all models, although scores remained within acceptable range for N95 respirators.
Zhao 2020 [85]	UVC (254 nm from mercury lamps or 265 nm from LED) 1 cycle of either 1000 mJ/cm^2^ or 10,000 mJ/cm^2^	3M 1860 and Moldex 1500	Negligible effects on particle filtration efficiency irrespective of dose. Negligible effects on polymer structure, morphology, surface hydrophobicity, or pressure drop and tensile strength of respirator materials, irrespective of applied dose.

LED, light-emitting diodes; UVC, ultraviolet light C. (!) Studies only available as preprint at the time of manuscript preparation and therefore yet to be peer reviewed.

**Table 7:** Studies reporting on the effects of heat treatment on filtering facepiece respirators (FFRs).

Study	Treatment Details	FFRs	Key Findings
Viscusi 2007 [41]	Dry heat in laboratory oven Treatment 1: 80 °C for 60 min Treatment 2: 160 °C for 60 min In both, FFRs were turned over at 30 min	1 unidentified N95 FFR model	At 80 °C, there was a small increase (negligible) in average filter particle penetration. At 80 °C, there were no visible changes after 60 min. At 160° C, FFRs largely melted.
Viscusi 2009 [77]	Dry heat in laboratory oven Treatment for 1 h at 80, 90, 100, 110, and 120 °C	Not identified by the authors, but included 3 N95 FFRs and 3 surgical N95 respirators	Results are difficult to interpret, but it seems that the models tested maintained their expected aerosol filtration efficiency at 80 °C and 90 °C, without any evident signs of damage.
Bergman 2010 [78]	3 cycles of moist heat incubation 30 min incubation at 60 °C, 80% RH in laboratory incubator After 1st incubation, samples were removed from incubator and air-dried overnight. After 2nd and 3rd incubations, samples were removed from incubator and air-dried for 30 min using a fan	Not identified by the authors, but included 3 N95 FFRs and 3 surgical N95 respirators	Heat-treated samples maintained required levels of filter aerosol penetration and filter airflow. Treatment caused all samples of one FFR model to have partial separation of the inner foam nose cushion from the FFR.
Bergman 2011 [79]	Moist heat incubation 3 cycles, 15 min at 60 °C, 80% RH	3M 1860, 3M 1870, and Kimberly Clark PFR95-270	There were no significant changes in FFR fit. 3M 1870 samples experienced a slight separation of the inner foam nose cushion (some to a lesser or greater degree) from the FFR body, but multiple treatments did not appear to increase the level of separation compared to a single treatment.
Viscusi 2011 [80]	Moist heat incubation 30 min at 60 °C, 80% RH	3M 8000, 3M 8210, Moldex 2200, 3M 1860, 3M 1870, and Kimberly Clark PFR95–270	For two models (3M 8210 and Moldex 2200), there was a reduction in fit; for one model (3M 1860), there was a small increase in odor response; but both effects were deemed to be negligible. 3M 1870 samples experienced a slight separation of the inner foam nose cushion (some to a lesser or greater degree) from the FFR body. Authors concluded that moist heat incubation unlikely to lead to significant changes in fit, odor detection, comfort, or donning difficulty.
Lore 2012 [81]	Moist heat incubation Uncertain temperature, but likely 65 °C for 20 min, unknown RH	3M 1860s, 3M 1870	There was no significant decrease in filter performance.
Anderegg 2020 [92]	Moist heat treatment for 30 min at 85 °C and 60–85% RH (there were additional 10 min at start until temperature and RH reach target levels)	3M 1860, 3M 1870, 3M 8210 Plus, HKYQ N95, and Chen Heng V9501 KN95	All FFRs passed particle filtration efficiency testing after 5 heat treatment cycles. All 3M FFRs passed quantitative fit testing after 5 heat treatment cycles. Chen Heng V9501 KN95 and HKYQ N95 FFRs failed fit testing before any treatment.
Daeschler 2020 [68]	5, 10, or 15 heat treatment cycles depending on test type Dry heat: 60 min at 70 °C and 0% RH Moist heat: 60 min at 70 °C and 50% RH	3M 8110s, 3M 9105s, 3M 8210, and 3M 1860s	Microstructural analysis of N95 filter layer (max 10 cycles at 0% and 50% RH)—no effect on diameter of filter fibers. Fit testing (max 15 cycles at 0% and 50% RH)—all FFRs passed tests. Particle filtration efficiency (max 10 cycles at 0% and 50% RH)—all FFRs passed tests. Breathing resistance (max 10 cycles at 0% and 50% RH)—all FFRs passed tests.
Doshi 2020 [93](!)	Moist heat treatment on a stove: ≥40 min at 65–80 °C and ~40–60% RH	Unknown Kimberly Clark model	Rudimentary testing showing no effect on particle filtration efficiency after 5 cycles. Primary aim of the study seems to have been to demonstrate that is feasible to heat treat N95 FFRs at home using kitchen utensils on a gas stove.
Fischer 2020 [39]	Dry heat (oven) at 70 °C, unknown RH Up to 3 cycles of 2 h of wear and likely 60 min of treatment	3M 9211+	There was a progressive reduction in filtration performance of respirators, which was below acceptable range after 3rd cycle.
Harskamp 2020 [88]	Autoclave 34 min cycle: 12 min pre-heating, 17 min steam treatment at 121 °C, and 5 min drying Up to 3 cycles	FFP2: 3M 1862+, 3M 9322+, Maco Pharma ZZM002, and San Huei 2920V FFP3: Safe Worker 1016	50% of FFP3 respirators were deformed and failed seal checks; all other respirators were intact upon inspection. The 3M 1862+ was the only respirator that continued to perform within the required range after 3 treatment cycles. All other respirators had particle filtering efficiency affected after one treatment cycle, performing below the required range (particularly for smaller particles—0.3 μm), with the magnitude of the reduction in performance varying between models.
Li 2020 [94]	20 cycles of 30 s 100 °C steam treatments (inside a steamer)	3M 1860	Methods lacking details, and amongst other things, unclear whether there was a cool down period between cycles (due to short duration). Authors concluded that 20 cycles “did not affect fit testing performance”, but few details provided.
Liao 2020 [44]	Dry heat: up to 50 cycles of 30 min at 75 °C, unknown RH Low RH heat: up to 50 cycles of 20 min at 85 °C and 30% RH Moist heat: up to 20 cycles of 20 min at 85 °C and either 70% or 100% RH Steam treatment: 10 min with water vapor (i.e., ~100 °C)	15 x 15 cm pieces of meltblown fabric, described as the most important layer of N95 FFRs	Dry heat: no appreciable decrease in filtration efficiency after 50 cycles (i.e., 1500 min). Low RH heat: unaffected filtration efficiency of fabric after 50 cycles (i.e., 1000 min). Moist heat: unaffected filtration efficiency of fabric after 20 cycles (i.e., 400 min). Steam treatment: no change in filtration efficiency after 3 cycles, but drop in efficiency (from ~97% to ~85%) after 5 cycles, explained by the authors as due to loss of static charge of the fibers.
Liao 2020 [44]	Low RH heat: up to 20 cycles of 20 min at 85 °C and 30% RH Moist heat: up to 20 cycles of 20 min at 85 °C and 100% RH	3M 8210, 4C Air KN95, ESound KN95, and Onnuripan KF94	All models tested retained filtration efficiency >95% after 20 treatment cycles (i.e., 400 min).
Loh 2020 [95](!)	Dry heat at 65 or 86 °C, 34–56 min per cycle (variable) Only 1 treatment cycle per respirator	FFP2–3M 9320+ and 3M 8810 FFP3–3M 9332+, 3M 1863+, 3M 1873V+, 3M 8833, 3M 8835, Alpha S-3V, and Honeywell 5321	There was a reduction in fit observed for all masks after one cycle, but the rate of reduction was highly variable, and most passed fit testing. Study was not standardized and it is difficult to interpret, but key message was variability between models. Note that one respirator failed the fit testing before any treatment.
Ou 2020 [43]	Dry heat (oven): 30 min at 77 °C, unknown RH Steam treatment: 30 min with water vapor (i.e., ~100 °C)	3M 8210	10 cycles of dry heat or steam treatment had negligible effects on particle filtration efficiency. Dry heat treatment had no effect on the N95 fit. 5 cycles of steam treatment led to failure in fit testing, with evidence of some effect appearing after just one cycle.
Price 2020 [84](!)	Dry heat (oven): 30 min at 75 °C 5 cycles, interspaced with 10 min cool-down periods at room temperature	3M 8200, 3M 8210+, 3M 8511, 4C AIR KN95, and Jackson R20	5 cycles of heat treatment had a negligible effect on fit testing performance of all 5 mask models tested.
Tsai 2020 [22]	Dry heat at 92 °C, unknown RH Moist heat at 92 °C, 85% RH Treatment duration not provided, but context suggests 15 min	One unidentified N95 respirator	Minimal information provided, other than basic data showing no effect on particle filtration efficiency after 4 cycles (each 24 h apart) of either dry or moist heat treatment.
Xiang 2020 [96]	Dry heat at 70 °C (electric oven), unknown RH Single continuous treatment course of 1, 2, or 3 h	3M 1860	No reported change in “shape”; no details provided of fit testing results but authors imply that respirators were largely unaffected after 3 h treatment. Minor reduction in filtration efficiency for bacterial aerosols (from 99% to 97% after 3 h) but still within acceptable range (i.e., ≥95%).
Yim 2020 [97](!)	Dry heat at 70 °C (oven), unknown RH Single continuous treatment up to 90 min	3M 1860 and Yomasi KN95	Yomasi KN95 model had filtration efficiency testing <95% even before testing (~83%). Negligible effects of 90 min at 70 °C on filtration efficiency.

RH, relative humidity. (!) Studies only available as preprint at the time of manuscript preparation and therefore yet to be peer reviewed.

**Table 8:** Summary of findings from the studies reporting on the effects of dry heat treatment (☼) and moist heat treatment (≈) on filtering facepiece respirators (FFRs).

	Heat Treatment Temperature									
Cumulative Treatment Time	60 °C	65 °C	70 °C	75 °C	77 °C	80 °C	85 °C	90 °C	92 °C	100 °C
10 min										≈[94]
20 min		≈[81]								
30 min	≈[80] ^1^									≈[44]
45 min	≈[79] ^1^									
50 min							☼[95] ^2^			
56 min		☼[95] (!)								
60 min						☼[41,77]		☼[77]	☼[22] ^3^ ≈[22] ^3^	
90 min	≈[78] ^1^		☼[97] (!)							
120 min			☼[39]							
150 min				☼[84]			≈[92]			
180 min			☼[96]							
200 min			≈[93](!) ^4^							
300 min					☼[43]					
400 min							☼[44] ^5^ ≈[44]			
600 min			☼[68](!) ≈[68](!)							
1000 min							☼[44] ^5^			
1500 min				☼[44]						

Cells contain the citations for a given study, with the corresponding temperature tested and the reported cumulative treatment time after which FFR performance/fit remained within the acceptable range. Studies where temperatures tested were above 100 °C have been excluded. ^1^ There was, however, separation of inner foam in one FFR model. ^2^ Temperature was 86 °C but rounded for simplicity. ^3^ Total time is an estimate. ^4^ Average temperature and length of exposure are rough estimates. ^5^ Low-moisture heat (30% RH). (!) Studies only available as preprint at the time of manuscript preparation and therefore yet to be peer reviewed.
